# Spectral Regulation in Cs_2_PtCl_6_ Double Perovskite via Low-Temperature and High-Pressure Engineering for Advanced Optical Thermometry and Manometry

**DOI:** 10.1021/acsami.5c12347

**Published:** 2025-08-22

**Authors:** Zhiyu Pei, Marcin Runowski, Xuanyu Ge, Przemysław Woźny, Laihui Luo, Peng Du

**Affiliations:** † School of Physical Science and Technology, 47862 Ningbo University, 315211 Ningbo, Zhejiang, China; ‡ Faculty of Chemistry, 467899 Adam Mickiewicz University, Uniwersytetu Poznańskiego 8, 61-614 Poznań, Poland

**Keywords:** Optical manometry, Optical thermometry, Luminescence, Double perovskite, Pressure and temperature sensing

## Abstract

To build a bifunctional luminescent platform for optical thermometry and manometry, the Cs_2_PtCl_6_ double perovskite was synthesized. Upon 468 nm excitation, the designed compound emitted bright red light centered at ≈675 nm, originating from the ^3^T_1g_,^3^T_2g_ → ^1^A_1g_ transition of Pt^4+^, whose fluorescence intensity and bandwidth are highly temperature dependent. Moreover, the temperature-dependent decay curves of the developed Cs_2_PtCl_6_ double perovskite were measured to explore its application in optical thermometry, of which its maximum relative sensor sensitivity is 1.21% K^–1^, with the operation range of 100–310 K. Furthermore, the impact of pressure on the phase structure and luminescence behaviors of the Cs_2_PtCl_6_ double perovskite was also discussed. The pressure-dependent Raman spectra clarified that the synthesized compound possesses a stable phase structure at high pressure. Furthermore, Cs_2_PtCl_6_ double perovskite exhibits distinct spectral blue-shift at high pressure. Importantly, as pressure increases (i.e., 0–5.67 GPa), the emission band centroid and full width at half-maximum (fwhm) are both linearly dependent on pressure, resulting in high pressure sensitivities of dλ/d*p* = 7.19 nm/GPa and d*fwhm*/d*p* = 4.95 nm/GPa, respectively. Our finding manifests that the luminescence characteristics of the Cs_2_PtCl_6_ double perovskite can be efficiently regulated via low temperature and high pressure, enabling its applications in optical thermometry and manometry.

## Graphical abstract

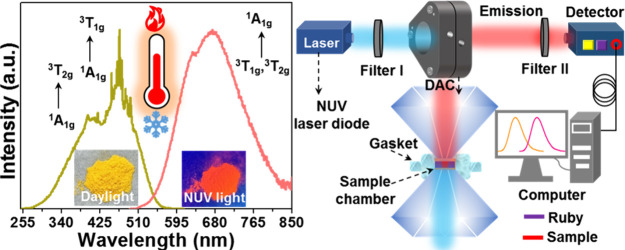

## 1 Introduction

Pressure and temperature, as two fundamental thermodynamic parameters, not only impact the biological, chemical, and physical properties of materials and living creatures but also play significant roles in scientific research and industrial manufacturing, and thus, their accurate monitoring is highly important. That is why noncontact remote measurements have attracted considerable attention on account of their advantages of real-time feedback, high spatial resolution, noninvasive detection, etc.^1,2^ Currently, to satisfy the requirement of modern scientific research and industrial technologies, a diamond anvil cell (DAC) has been widely adopted to generate an isotropic hydrostatic pressure. Moreover, through the utilization of the optical pressure sensing method, the generated static high pressure in a DAC can be remotely monitored, mainly because of the high transparency of diamonds.^3^ At present, using the emission band centroid as a manometric parameter, ruby (Al_2_O_3_:Cr^3+^) is commonly adopted to detect the pressure in a DAC; nevertheless, it suffers from low pressure sensitivity (dλ/d*p*) of 0.365 nm/GPa.^4^ To settle this drawback, several different types of luminescent materials with high sensitivities, such as NaY_9_(SiO_4_)_6_O_2_:Mn^2+^ (dλ/d*p* = 7.00 nm/GPa), Ca_5_(BO_3_)_3_F:Bi^3+^ (dλ/d*p* = 4.76 nm/GPa), Sr_2_(MgAl_5_N_7_):Eu^2+^ (dλ/d*p* = 5.07 nm/GPa), Li_4_SrCa­(SiO_4_)_2_:Eu^2+^ (dλ/d*p* = 5.19 nm/GPa), etc.,^5–8^ were developed for remote pressure sensing. Clearly, searching for novel luminescent material is a reasonable and efficient strategy approach to realize highly sensitive and real-time optical pressure monitoring.

Aside from pressure, temperature monitoring has been also widely studied, of which substantial interests are gained in optical thermometry that takes advantage of thermoresponsive spectroscopic behaviors including fluorescence intensity ratio (FIR) of thermally coupled levels (TCLs) of rare-earth ions, decay time, bandwidth, etc., of various luminescent materials.^9–13^ Over the last decades, most of the reported optical thermometers relied on the temperature-dependent FIR value of TCLs of rare-earth ions, whereas their relative sensor sensitivities (*S*_*r*_) are highly dependent on the energy separation (Δ*E*) of TCLs, i.e., *S*_*r*_ = Δ*E*/*kT* ^2^ (*k* and *T* are Boltzmann constant and temperature, respectively),^14–16^ resulting in unsatisfied *S*_*r*_ value, which is not beneficial for their vivid applications. Moreover, the TCLs-based optical thermometry mainly focused on high-temperature sensing, while the study on low-temperature detection is still insufficient. In this regard, the interest in investigating the thermometric properties based on non-TCLs, bandwidth, and decay time of luminescent materials is increasing. Hence, recently, several new optical thermometers, such as NaSrY­(MoO_4_)_3_:Er^3+^/Tm^3+^/Yb^3+^ (*S*_*r*_ = 0.95% K^–1^), Ba_3_LuGa_2_O_7.5_:Bi^3+^ (*S*_*r*_ = 2.21% K^–1^), Ca_3_La_2_W_2_O_12_:Mn^4+^/Dy^3+^ (*S*_*r*_ = 1.551% K^–1^), Lu_2_CaMg_2_Ge_3_O_12_:Yb^3+^/Nd^3+^/Er^3+^ (*S*_*r*_ = 2.62% K^–1^), and so forth, were developed,^17–20^ whose thermometric properties were found to be significantly impacted by host composition. In spite of these achievements, more efforts are still required to further explore and regulate the temperature-sensing capacities of luminescent materials.

To date, lead-free double perovskites with the chemical formula of A_2_BX_6_, especially, chlorides, have been extensively designed as luminescent materials owing to their intense broadband emission from self-trapped excition, large Stokes shifts, good stability, etc.^21–23^ Through the codoping of Er^3+^ and Yb^3+^, Zhang et al. stated that the Cs_2_NaInCl_6_:Er^3+^/Yb^3+^ crystals can emit pure green upconversion emission, allowing its application in optical thermometry.^24^ It was reported that the Cs_2_ZrCl_6_ double perovskite can emit broad deep-blue emission, and it was the promising luminescent candidate for various applications, such as lighting, X-ray imaging, temperature sensing, etc.^25^ As for the Cs_2_HfCl_6_ double perovskite, its intrinsic luminescence also pertains to blue emission, and it can be manipulated via doping engineering, resulting in the applications of white light-emitting diodes, optical thermometry, etc.^26,27^ Moreover, Xiao et al. pointed out that the Cs_2_PtCl_6_ double perovskite can not only exhibit bright red light, but is also suitable for designing high-quality white light-emitting diodes.^28^ Furthermore, our recent work also indicates that the luminescence of the Cs_2_Ag_0.6_Na_0.4_InCl_6_:Bi^3+^ double perovskite can be regulated via applying pressure, where its emitting color changes from yellow to blue with increasing the pressure up to 4 GPa, contributing to an ultrahigh pressure sensitivity of 112 nm/GPa.^29^ The previous progress implies that the A_2_BX_6_ double peroveskites with admirable luminescence performances are multifunctional platforms for diversified applications. However, to the best of our knowledge, the impact of both low temperature and high pressure on the luminescence properties of A_2_BX_6_ double perovskites is still lacking, requiring exploration of their applications in optical thermometry and manometry. Moreover, as mentioned above, the mentioned sensor operates only in the very low pressure range, limiting its utility in several applications.

For the sake of circumventing these shortages, Cs_2_PtCl_6_ was selected as the research target, of which its crystal structure, morphology, elemental composition, and luminescence features were analyzed in detail. Furthermore, based on the determined low-temperature decay time of the Cs_2_PtCl_6_ double perovskite, its thermometric properties were discussed, and the maximum *S*_*r*_ value was calculated to 1.21% K^–1^. Through investigation of the Raman spectra, the structure stability of the resulting sample at high pressure was studied. Most importantly, the influence of pressure on the luminescence characteristics of the synthesized product was studied via examining its pressure-dependent emission spectra in the range of 0–5.67 GPa. A significant pressure-triggered spectral blue-shift was observed in the Cs_2_PtCl_6_ double perovskite, contributing to the high pressure sensitivity of dλ/d*p* = 7.19 nm/GPa, when the emission band centroid was adopted as the manometric parameter. These results demonstrate that the luminescence properties of the Cs_2_PtCl_6_ double perovskite can be regulated via low-temperature and high-pressure engineering, enabling its promising applications in optical thermometry and manometry.

## 2 Experimental Section

### 2.1 Preparation of Cs_2_PtCl_6_ Double Perovskite

Via utilization of the hydrothermal method, a Cs_2_PtCl_6_ double perovskite was prepared. According to the stoichiometric ratio, 2 mmol of CsCl (99.95%) and 1 mmol of PtCl_6_ (99.95%) were weighted and then put into an autoclave (25 mL). Subsequently, 3 mL of HCl (37%) was added to the above mixture. After stirring for 10 min, the solution was kept in an oven and heated at 200 °C for 12 h. Finally, the final product was washed with ethanol two times and then dried at 80 °C for 2 h.

### 2.2 Characterizations

The phase structure and elemental composition of the synthesized double peroveskite were characterized through an X-ray diffractometer (Bruker D8 Advance, Cu Kα irradiation) and an X-ray photoelectron spectrometer (Thermo ESCALAB 250XI), respectively. A field-emission scanning electron microscope (SEM; HITACHI SU3500) attached with an energy-dispersive X-ray (EDX) spectrometer and transmission electron microscope (TEM; JEM-2100F, JEOL) were employed to explore the morphological characterizations of the studied material. The emission and excitation spectra of the Cs_2_PtCl_6_ double perovskite were measured by a fluorescence spectrometer (Edinburgh FS5). As for the excitation power-dependent emission spectra, they were recorded with a fluorescence spectrometer (Ocean Optics USB2000+).

To investigate the Raman and emission spectra of the compressed Cs_2_PtCl_6_ double perovskite under extreme conditions of pressure, a diamond anvil cell (DAC) assembly was adopted. A tiny amount of the synthesized double perovskite powder material was placed in a hole (≈150 μm) drilled in a stainless steel gasket, whose thickness was 250 μm. During the measurement process, the mixture of methanol/ethanol/water (i.e., volume ratio was 16:3:1) was employed as the pressure transmission medium, and the generated pressure in the DAC was calibrated through observing the spectral redshift of ruby *R*_1_ emission line. Upon 375 nm excitation, the pressure-dependent emission spectra were tested by means of a spectrometer (Andor Shamrock 500i) equipped with a silicon CCD camera detector. Furthermore, the pressure-related Raman spectra were detected by a confocal micro-Raman system (Renishaw InVia), where a 532 nm laser diode was utilized as the excitation source.

## 3 Results and Discussion

The crystal structure and atomic locations of the Cs_2_PtCl_6_ unit cell are shown in Figure 1(a), of which the [PtCl_6_]^2–^ octahedra occupy the center positions of corners and faces. The recorded X-ray diffraction (XRD) pattern presented in Figure S1 indicates that the resulting sample possesses cubic phase (PDF#07-0200), which is further confirmed by the Rietveld XRD refinement result, as depicted in Figure 1(b). According to the refinement result, one knows the lattice parameters of the synthesized Cs_2_PtCl_6_ double perovskites are *a* = *b* = *c* = 10.2131 Å and *V* = 1065.301 Å^3^. For the sake of exploring the elemental compositions of the final product, an X-ray photoelectron spectroscopy (XPS) measurement was carried out. As disclosed in Figure 1(c), the studied double perovskite is composed of Cs, Pt, and Cl elements. Herein, the high-resolution XPS spectrum of Cs^+^ 3d consists of two intense bands with the binding energies of 723.2 and 737.2 eV corresponding to Cs^+^ 3d_5/2_ and Cs^+^ 3d_3/2_, respectively (see Figure S2­(a)).^30^ Moreover, two peaks at 74.8 and 77.1 eV, which are assigned to Pt^4+^ 4f_5/2_ and Pt^4+^ 4f_7/2_, respectively, are observed in the high-resolution XPS spectrum of Pt^4+^ (Figure S2­(b)).^31^ The high-resolution XPS spectrum of Cl^–^ 2p shown in Figure S2­(c) exhibits an asymmetric peak, and it can be divided into two parts at 198.2 and 199.8 eV pertaining to Cl^–^ 2p_1/2_ and Cl^–^ 2p_3/2_, respectively.^30^ On the other hand, both the SEM and TEM images confirm that the prepared double perovskite is made up of microparticles, as displayed in Figure 1(d) and (e), respectively. Furthermore, the high-resolution TEM image exhibits distinct lattice fringes, and their adjacent distance is revealed to be 3.66 Å, which corresponds to the (220) plane of the cubic Cs_2_PtCl_6_ (PDF#07–0200), as shown in Figure 1(f). Furthermore, bright dots are detected in the selected area electron diffraction pattern (Figure 1(g)), manifesting the single-crystal nature of the designed compounds. Aside from the XPS, the elemental composition in the synthesized samples is also verified by the EDX spectrum (Figure 1(h)). In addition, the elements presented in the designed compounds are uniformly distributed throughout the whole particles, as confirmed by the elemental mapping results (Figure 1(i)–(l)). These results suggest that the Cs_2_PtCl_6_ double material has been successfully synthesized.

**Figure 1:**
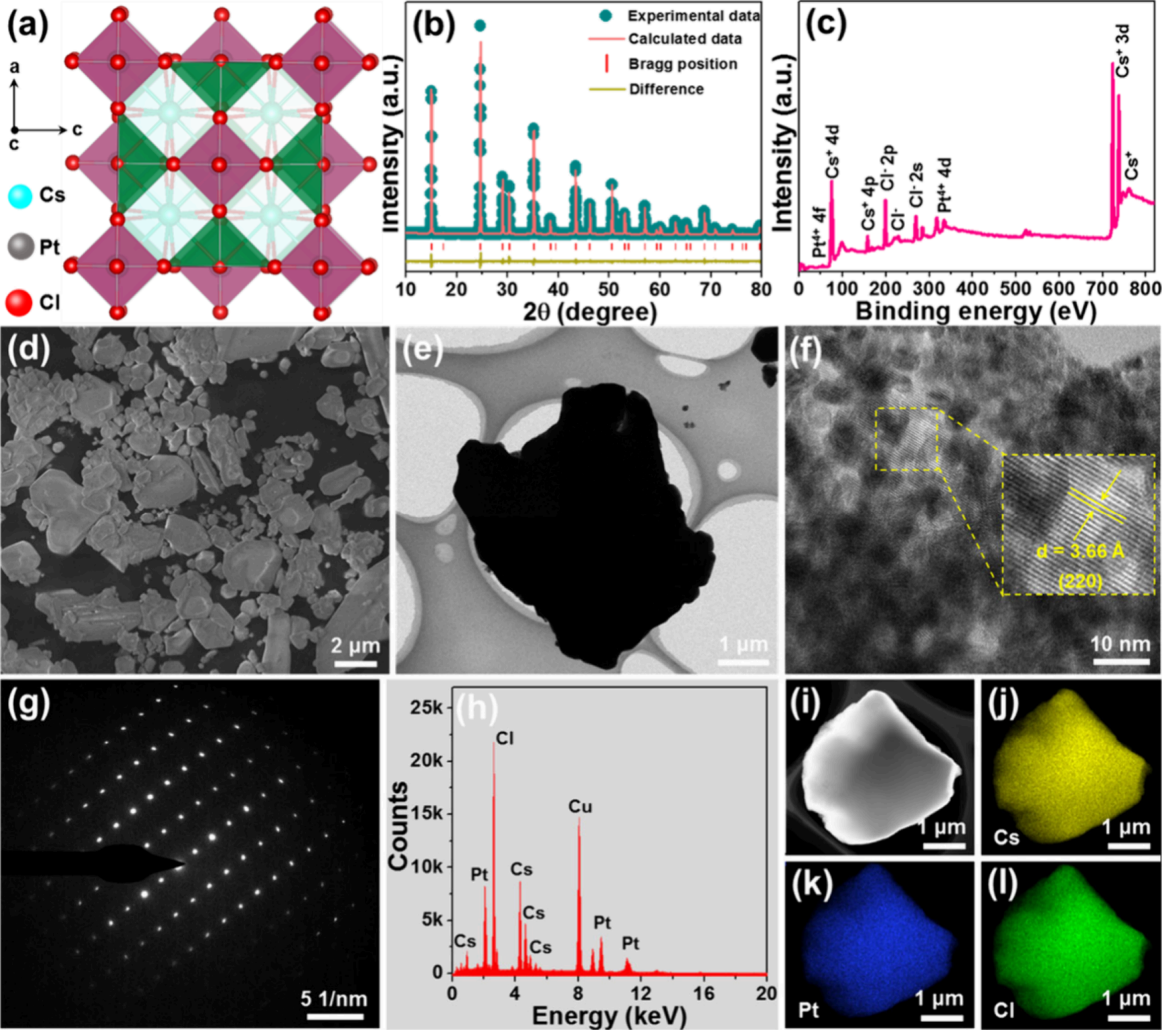
(a) 3D representation of the Cs_2_PtCl_6_ crystal structure. (b) Rietveld refinement of the experimental XRD pattern, (c) full range XPS spectrum, (d) SEM image, (e) TEM image, (f) high-resolution TEM image, (g) SAED pattern, (h) EDX spectrum, and (i)–(l) elemental mapping of the Cs_2_PtCl_6_ double perovskite.

To study the luminescence properties of the Cs_2_PtCl_6_ double perovskite, its excitation and emission spectra were measured and are demonstrated in Figure 2(a). The recorded excitation spectrum, monitored at 675 nm, consists of two bands at around 410 and 468 nm, which are assigned to the transitions of Pt^4+^, from ^1^A_1g_ to ^3^T_2g_ and ^3^T_1g_ levels, respectively, within the Pt^4+^ 5d t_2g_^6^e_g_^0^ electronic configuration, in the octahedral ligand field.^28^ The emission spectrum, measured at 468 nm excitation, shows a broad band with a central wavelength of 675 nm, originating from the ^3^T_1g_,^3^T_2g_ → ^1^A_1g_ transition (Figure 2(a)). Moreover, the resulting samples can emit bright red light (see Figure 1(a)), with a quantum efficiency of 16.5%, when excited at 468 nm (Figure S3). For the aim of investigating the origin of the observed emission in the Cs_2_PtCl_6_ double perovskite, its excitation power density-dependent emission spectra were tested and are demonstrated in Figure 2(b). Clearly, the fluorescence intensity exhibits an upward tendency as excitation power density increases, and the saturation phenomenon does not occur. Herein, the relation between fluorescence intensity and excitation power density is linear (see Figure 2(c)), suggesting that the emission in Cs_2_PtCl_6_ double perovskite cannot be attributed to the permanent defect, since the emission of permanent defect is limited and will saturate at high excitation power density.^32,33^ Furthermore, the decay time of the emission at 675 nm is found to be 26.5 μs (see Figure S4), excluding the free exciton emission, since its decay time should be within the nanosecond scale.^34^ Additionally, the temperature-dependent emission spectra of the Cs_2_PtCl_6_ double perovskite were measured and are depicted in Figure 2(d). With an increase in the temperature, it is found that the peak width also increases (i.e., from 271 to 392 meV), aside from deteriorated emission intensity. Note that the thermally enhanced electron–phonon coupling can be responsible for the broadened emission band.^24^ It is well established that the temperature-related full width at half-maximum (fwhm) value can be quantitatively fitted via utilizing the following function:^35^

1 $$\mathrm{fwhm}=2.36\sqrt{S}\hbar \omega \sqrt{\coth\left(\frac{\hbar \omega }{2kT}\right)}$$

where ℏω denotes the phonon energy, *S* is the Huang–Rhys factor, and *k* (i.e., *k* = 8.629 × 10^–5^ eV/K) describes the Boltzmann coefficient. According to the fitting results (Figure 2(e)), the resulting ℏω and *S* values are 15.1 and 34.6, respectively. Since the calculated *S* value is much larger than 5, we can conclude that the electron–phonon coupling in the Cs_2_PtCl_6_ double perovskite is strong.^29^ On the other hand, for the purpose of clarifying the thermal quenching of luminescence in the Cs_2_PtCl_6_ double perovskite, the corresponding exciton binding energy (*E*_*a*_) was estimated by means of the following formula:^36,37^

2 $$I=\frac{I_{0}}{1+A\;\exp\left(-E_{a}/kT\right)}$$

where the fluorescence intensities at initial and measured temperatures are described by *I*_0_ and *I*, respectively, and *A* is constant. Through analyzing the experimental data, it was found that the *E*_*a*_ value is determined to be approximately 114 meV, as illustrated in Figure 2(f).

**Figure 2:**
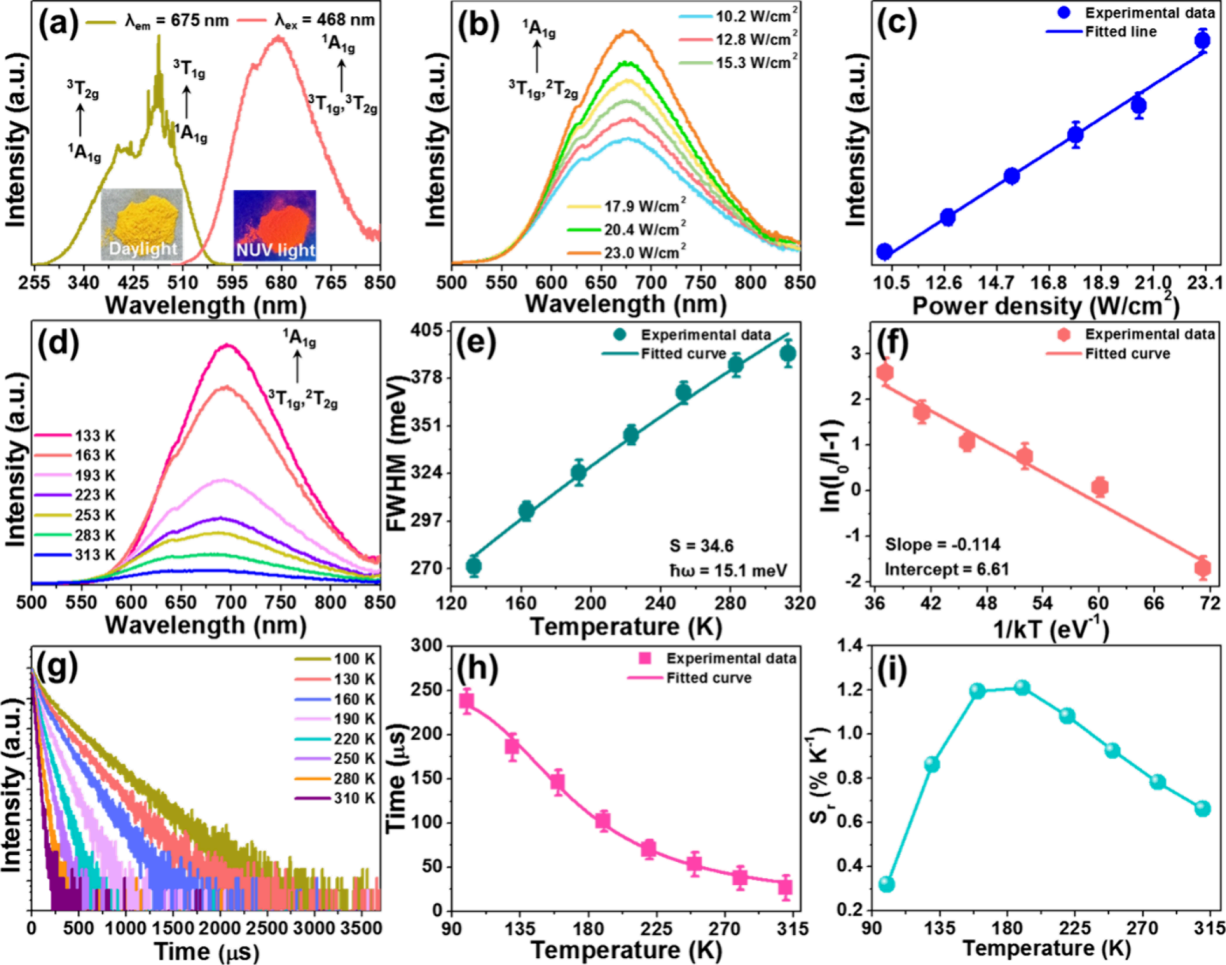
(a) Excitation and emission spectra of the Cs_2_PtCl_6_ double perovskite; the inset shows the optical images of the resulting compound in daylight and under UV light irradiation. Laser power density dependent (b) emission spectra and (c) the corresponding fluorescence intensities of the Cs_2_PtCl_6_ double provskites as a function of laser power density. (d) Emission spectra and (e) fwhm values of the Cs_2_PtCl_6_ double perovskite as a function of temperature. (f) Plot of ln­(I_0_/I – 1) vs 1/*kT* for the Cs_2_PtCl_6_ double perovskite. Temperature-dependent (g) decay curves and (h) the determined excited state lifetimes for the Cs_2_PtCl_6_ double perovskite. (i) The calculated *S*_*r*_ values as a function of temperature.

In an attempt to explore the feasibility of the Cs_2_PtCl_6_ double provskites for optical thermometry, the temperature-dependent decay curves (i.e., λ_ex_ = 468 nm and λ_em_ = 675 nm) were examined in the range 100–310 K and are displayed in Figure 2(g). Herein, the average excited state lifetime (i.e., τ) was estimated by means of the following function:

3 $$\tau =\frac{\int I\left(t\right)\times tdt}{\int I\left(t\right)\times dt}$$

where the fluorescence intensity at time *t* is labeled by *I*(*t*). Evidently, as temperature arises (i.e., 100–310 K), the τ value decreases sharply; that is, the lifetime shortens from 237.6 to 26.5 μs (see Figure 2(h)). On the basis of previous literature data,^38^ one knows that the temperature-dependent lifetime should meet the following relation:

4 $$\tau =\frac{\tau_{0}}{1+A\;\exp\left(-\Delta E/kT\right)}$$

where τ_0_ and τ are the decay times at *T* = 0 K and *T*, respectively, and *A* is a coefficient. Through fitting the calculated data (Figure 2(h)), it was found that the relation between the lifetime and temperature can be presented as *τ =* 244.1/[1 + 69.9 exp­(−733.3/*T*)]. For the sake of quantitative evaluation of the temperature sensing capacities of the Cs_2_PtCl_6_ double perovskite, we determined the relative temperature sensitivity (*S*_*r*_) values, according to the following equation:^9,35,38^

5 $$S_{r}=\left|\frac{1}{\tau }\frac{d\tau }{dT}\right|\times 100\%$$

Accordingly, the determined *S*_*r*_ values within the temperature range 100–310 K are depicted in Figure 2(i). As disclosed, the *S*_*r*_ value increases first and then decreases as temperature arises, and its maximum value is 1.21% K^–1^ at 190 K. Importantly, the calculated *S*_*r*_ value is comparable with previously developed lifetime-based optical thermometers, such as Ca_3_La_2_W_2_O_12_:Mn^4+^/Dy^3+^ (*S*_*r*_ = 1.953% K^–1^), Cs_2_ZrCl_6_:Re^4+^ (*S*_*r*_ = 0.47% K^–1^), CsPb­(Cl/Br)_3_:Tb^3+^ (*S*_*r*_ = 0.94% K^–1^), and CaYGaO_4_:Cr^3+^ (*S*_*r*_ = 0.78% K^–1^),^19,35,38,39^ manifesting that the Cs_2_PtCl_6_ double perovskite is a promising candidate for the lifetime-based optical thermometry.

In view of the application of luminescent materials for optical manometry, they need to possess splendid structural stability at high pressure. Herein, to investigate the impact of high -pressure on the phase structure of the Cs_2_PtCl_6_ double perovskite, the pressure-dependent Raman spectra analysis was performed. Figure 3(a) depicts the normalized Raman spectra of the Cs_2_PtCl_6_ double perovskite during the compression process (i.e., 0–5.59 GPa). When *p* = 0 GPa, it can be seen that the recorded Raman spectrum consists of three bands, which initially situate at 171, 310, and 335 cm^–1^. Notably, as pressure elevates, the Raman bands shift to higher wavenumbers (higher energies) due to the shortened bond lengths (i.e., smaller interatomic distances) upon the compression process. Moreover, when the Cs_2_PtCl_6_ double perovskite undergoes the decompression process (i.e., pressure release from 5.59 to 0.54 GPa), it is clearly seen that the Raman peaks return to their initial states (i.e., shift to lower wavenumbers), as shown in Figure 3(b). Furthermore, through analyzing the relation between Raman peak centroids and pressure, one knows that the shift rates of these Raman modes initially located at 171, 310, and 335 cm^–1^ are 2.02, 6.52, and 377 cm^–1^/GPa, respectively, as presented in Figure 3(c)–(e). Aside from the linear spectral shift with increasing pressure, the Raman bands are slightly broadened, and the signal-to-noise ratio in the Raman spectra is deteriorated. These effects are triggered by the increased number of crystal defects and strains in the compressed material. The presented results manifest that the investigated material sample has a stable phase structure within the pressure range of at least 0–5.59 GPa.

**Figure 3:**
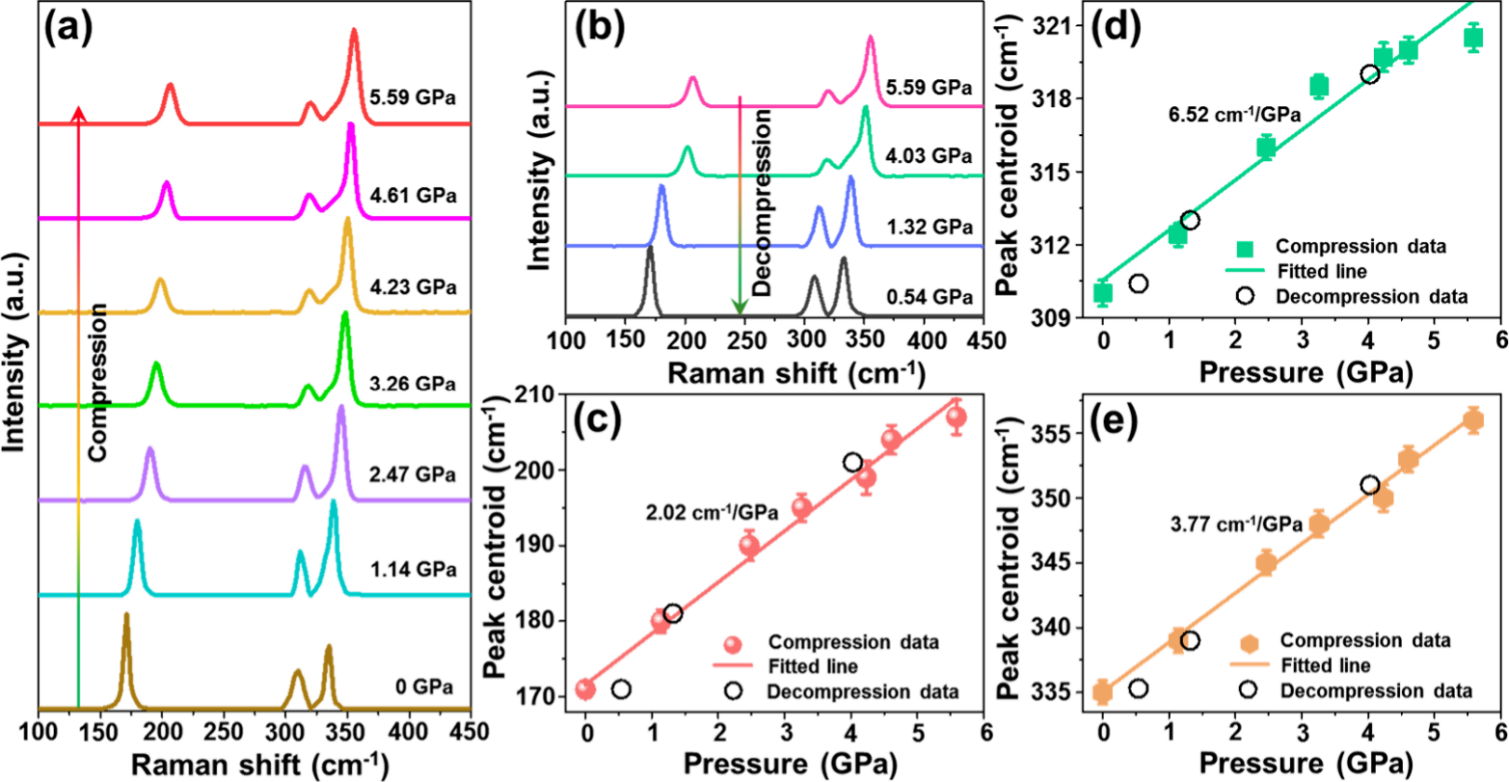
Pressure-dependent Raman spectra of the Cs_2_PtCl_6_ double perovskite in the range of (a) 0–5.59 GPa (i.e., compression process) and (b) 5.59–0.54 GPa (i.e., decompression process). (c–e) Raman peak centroids of the Cs_2_PtCl_6_ double perovskite as a function of pressure.

For the purpose of revealing the influence of pressure on the luminescence features of the material studied, a homemade measurement system was employed, as shown in Figure 4(a). The obtained (normalized) pressure-dependent emission spectra of the Cs_2_PtCl_6_ double perovskite, measured in the range 0–5.67 GPa (λ_ex_ = 375 nm), are depicted in Figure 4(b), of which the corresponding optical images are presented in Figure 4(c). Clearly, with increasing pressure, the resulting compound still exhibits a broadband emission, whose centroid shifts to shorter wavelengths (i.e., blue-shift), that is, from 677 to 638 nm, when the pressure elevates from 0 to 5.67 GPa (see Figure 4(d)). Owing to the distinct blue-shift of the emission band, the color coordinates of the Cs_2_PtCl_6_ double perovskite change from (0.615,0.382) to (0.563,0.426) as pressure arises from 0 to 5.67 GPa, as displayed in Figure 4(c) and Table S1. Note that although the color coordinates change gradually as pressure increases they still locate in the red region, and the brightness of the emitted colors decreases at elevated pressure due to quenching processes. Aside from the color coordinate, the correlated color temperature of the emission in the Cs_2_PtCl_6_ double perovskite is found to be dependent on the pressure; i.e., its value changes in the range of 1858–1710 K, as displayed in Table S1 (for calculation details; see Supporting Information). From previous reports,^29,40,41^ one knows that the decreased lattice relaxation energy (*E*_*LR*_) is mainly responsible for the blue-shift of the observed emission band. As is known, the relation between *E*_*LR*_ and fwhm can be expressed as $\mathrm{fwhm}\propto \sqrt{2E_{LR}kT}$ (where *k* is a Boltzmann constant).^40,41^ Since the values of *k* and *T* are constants, fwhm is proportional to *E*_*LR*_. It is worth noting that the determined fwhm values for the Cs_2_PtCl_6_ double perovskite significantly decrease with pressure (see Figure 4(e)), i.e. from ≈167 to 139 nm, which is associated with the decreasing *E*_*LR*_ value for the compressed material. Thereby, the broadband emission in the Cs_2_PtCl_6_ double perovskite presents a blue-shift phenomenon under high-pressure conditions. Moreover, the shortened bond length and contracted octahedrons, which are triggered by high pressure, may also contribute to the blue-shift of the emission band. Furthermore, when the designed compound undergoes the decompression process, the emission band centroid and fwhm return back to their initial states, as illustrated in Figure 4(d), (e), and Figure S5, implying that Cs_2_PtCl_6_ double perovskite has superior structural stability and reversibility, which ensures its utility in high-pressure luminescence manometry (optical pressure sensing). The determined pressure-dependent emission band centroids of the Cs_2_PtCl_6_ double perovskite are presented in Figure 4(d). Apparently, the relation between emission band centroid (λ) and pressure (*p*) is linear, and it can be expressed as λ = 675.81 – 7.19*p*. Thereby, when the emission band centroid is utilized as the manometric parameter, the pressure sensitivity (i.e., d*λ/*d*p*) of the Cs_2_PtCl_6_ double perovskite is 7.19 nm/GPa. Herein, the calculated d*λ/*d*p* value is not only almost 20 and 30 times higher than those for the commonly used optical manometers of Al_2_O_3_:Cr^3+^ (d*λ/*d*p* = 0.365 nm/GPa) and SrB_4_O_7_:Sm^2+^ (d*λ/*d*p* = 0.255 nm/GPa) but is also larger than the majority of the previously developed optical manometers, as listed in Table 1. Aside from the emission band centroid, its fwhm values are also found to be linearly dependent on pressure, and their relation can be expressed as fwhm = 165.52 – 4.95*p*. Consequently, the pressure sensitivity (i.e., d*fwhm*/d*p*) of the developed Cs_2_PtCl_6_ double perovskite is found to be 4.95 nm/GPa, when the fwhm is adopted as the manometric parameter. Notably, the calculated d*fwhm*/d*p* value is larger than most of those of the previously developed optical manometer, as displayed in Table 1. Note that although the previously developed optical manometers, i.e., Cs_2_Ag_0.6_Na_0.4_InCl_6_:Bi^3+^, Zn_2_GeO_4_:Mn^2+^ or Ca_2_Gd_8_(SiO_4_)_6_O_2_:Mn^2+^, exhibit higher sensitivities than the Cs_2_PtCl_6_ double perovskite (Table 1) their values are not fixed (i.e., the sensitivity values are dependent on pressure), and in the case of the Cs_2_PtCl_6_ double perovskite, both d*λ/*d*p* and d*fwhm*/d*p* are constants over the whole opearting pressure range. Such characteristics make the Cs_2_PtCl_6_ double perovskite a suitable candidate for high-sensitive optical manometry. Additionally, the cycling experiment results reveal that the broad-band emission can go back to its starting state after two compression–decompression cycles, as illustrated in Figure S6, further confirming the good stability and reversibility of the Cs_2_PtCl_6_ double perovskite. These achievements suggest that the Cs_2_PtCl_6_ double perovskite with high sensor sensitivities has promising applications in optical pressure sensing.

**Figure 4:**
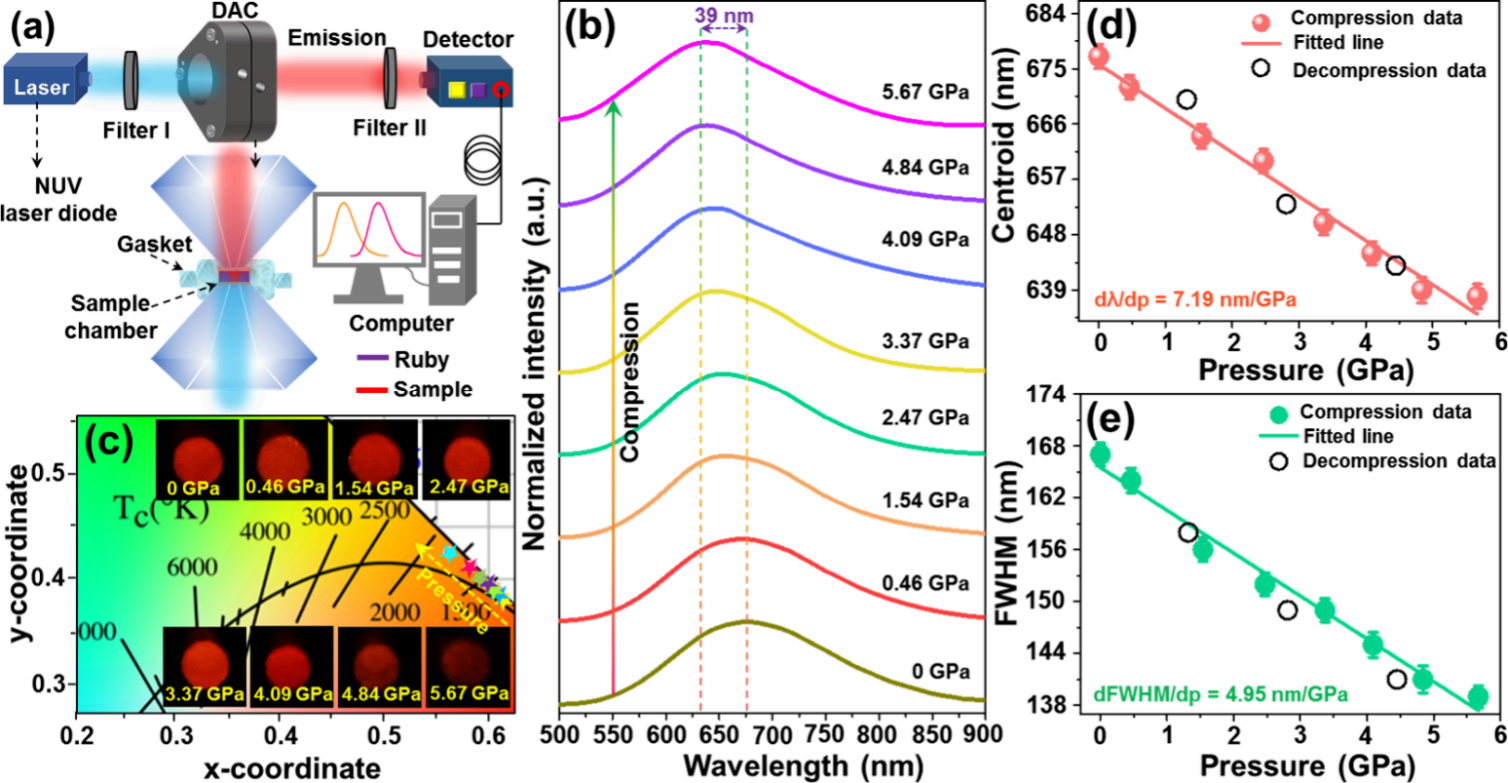
(a) Experimental setup used for the pressure-dependent luminescence measurements. (b) Emission spectra and (c) CIE chromaticity diagram for the Cs_2_PtCl_6_ double perovskite as a function of pressure; the inset of (c) shows the corresponding optical photographs of the material’s luminescence with increasing pressure. (d) Emission band centroid and (e) fwhm of the Cs_2_PtCl_6_ double perovskite as a function of pressure.

**Table 1:** Comparison of Manometric Properties of Cs_2_PtCl_6_ Double Perovskite with Other Developed Optical Manometers Operating in the Visible Range

	Sensing mode				
Compounds	dλ/d*p* (nm/GPa)	d*fwhm*/d*p* (nm/GPa)	Sensitivity value	Centroid (nm)	Ref
Ba_3_Lu(BO_3_)_3_:Ce^3+^	3.51	–	Fixed	485	^3^
Zn_2_GeO_4_:Mn^2+^	21.3	17.0	Pressure dependent	535.9	^4^
NaY_9_(SiO_4_)_6_O_2_:Mn^2+^	7.00	10.13	Pressure dependent	617.2	^5^
Sr_2_[MgAl_5_N_7_]:Eu^2+^	5.07	–	Fixed	649	^7^
Li_4_SrCa(SiO_4_)_2_:Eu^2+^	5.19	1.23	Fixed	584.7	^8^
La_3_Mg_2_SbO_9_:Mn^4+^	1.20	0.53	Fixed	690.5	^9^
Cs_2_Ag_0.6_Na_0.4_InCl_6_:Bi^3+^	112	31.5	Pressure dependent	600	^29^
Lu_2_Mg_2_Al_2_Si_2_O_12_:Eu^2+^	1.77	–	Fixed	473.8	^42^
Sr_8_Si_4_O_12_Cl_8_:Eu^2+^	9.69	–	Fixed	497.3	^43^
Ca_2_Gd_8_Si_6_O_26_:Ce^3+^	3.00	–	Fixed	396.8	^44^
Ca_2_Gd_8_(SiO_4_)_6_O_2_:Mn^2+^	7.25	–	Pressure dependent	592.2	^45^
Cs_2_PtCl_6_	7.19	4.95	Fixed	677	This work

## 4 Conclusion

Here, we show the development of a novel optical pressure sensor based on the emission of Cs_2_PtCl_6_ double perovskite, which is currently the second report in the world about double perovskite-based luminescent manometer, and our material can operate in a broader pressure range than the previously reported sensor of Cs_2_Ag_0.6_Na_0.4_InCl_6_:Bi^3+^ double perovskite. The Cs_2_PtCl_6_ double perovskite was designed to explore its applications in optical thermometry and manometry. Excited at 468 nm, the resulting compound has the ability to emit intense red emission at 675 nm, which belongs to the transition of ^3^T_1g_,^3^T_2g_ → ^1^A_1g_ of Pt^4+^. For the sake of investigating the thermometric properties of the studied sample, the temperature-related decay curves were measured. By using the lifetime as temperature indicator, one knows that the maximum *S*_*r*_ value of the Cs_2_PtCl_6_ double perovskite is 1.21% K^–1^, and it can be operated in the temperature range of 100–310 K. Moreover, to confirm the feasibility of the designed sample for optical manometer, the emission and Raman spectra were examined under extreme condition of pressure. The Raman spectra demonstrate that the Cs_2_PtCl_6_ double perovskite does not show a phase transition within the pressure range of our interest. Furthermore, as the pressure increases (i.e., 0–5.67 GPa), an evident blue-shift, i.e., from 677 to 638 nm, is observed in the emission band centroid. When the emission band centroid and fwhm are adopted as the manometric parameter, it is found that the pressure sensitivities of the Cs_2_PtCl_6_ double perovskite are 7.19 and 4.95 nm/GPa, which are all independent of pressure. These results suggest that the luminescence properties of the Cs_2_PtCl_6_ double perovskite can be regulated by low-temperature and high-pressure engineering, enabling its applications in optical thermometry and manometry. This work opens new horizons in development of novel, bifunctional pressure and temperature sensors, based on the rarely reported and exotic type of the double perovskites.

## Supplementary Material



## Notes

Calculation of the correlated color temperature, pressure-dependent color coordinate, XRD profile, XPS, quantum efficiency, and decay curve of Cs_2_PtCl_6_ double perovskite, Pressure-associated emission spectra of Cs_2_PtCl_6_ double perovskite during compression and decompression processes. (PDF)
