# Supplementary material for: Spectral Regulation in Cs2PtCl6 Double Perovskite via Low-Temperature and High-Pressure Engineering for Advanced Optical Thermometry and Manometry
Source: ACS Appl Mater Interfaces. 2025 Aug 22;17(35):49683–91. doi: 10.1021/acsami.5c12347 (PMC12412112; doi:10.1021/acsami.5c12347)
Supplement: Supplementary file 1 [file am5c12347_si_001.pdf]

Supporting Information

Spectral regulation in Cs_2PtCl_6 double perovskite via low-temperature and high-pressure engineering for advanced optical thermometry and manometry

Zhiyu Pei[a], Marcin Runowski[b*], Xuanyu Ge[a], Przemysław Woźny[b], Laihui Luo[a], Peng Du[a*]

[a]*School of Physical Science and Technology, Ningbo University, 315211 Ningbo, Zhejiang, China*

[b]*Adam Mickiewicz University, Faculty of Chemistry, Uniwersytetu Poznańskiego 8, 61-614 Poznań, Poland*

Corresponding authors:

E-mail: runowski@amu.edu.pl (M. Runowski); dp2007good@sina.com or dupeng@nbu.edu.cn (P. Du)

Calculation of the pressure-dependent CCT value

The correlated color temperature (CCT) values of the generated emissions in the Cs_2PtCl_6 double perovskite were calculated by means of the following functions [1]:

$$\mathrm{CCT} = -437n^3 + 3601n^2 - 6846n + 5514.31 \tag{S1}$$

$$n = (x - x_e)/(y - y_e) \tag{S2}$$

here $(x_e, y_e) = (0.3320, 0.1858)$ and (x, y) presents the color coordinates of the generated emissions. Through utilizing the aforementioned formuls and the calculated color coordinates, the pressure-dependent CCT values of the emission in the Cs_2PtCl_6 double perovskite were evalulated and presented in Table S1. It is clear that the CCT values are changed in the range of 1858-1710 K as pressure arises.

Reference

1. Zhang, M.; Guo, X.; Cui, R.; Zhang, J.; Deng, C. A thermally stable color-tunable white phosphor $CaGa_{0.5}Nb_{0.5}O3:Dy^{3+}/Sm^{3+}$ for warm WLEDs, *Ceram. Int.* **2025**, *51*, 9740-9752.

Table S1 Color coordinates and CCT value of the Cs_2PtCl_6 double perovskite as a function of pressure.

Pressure (GPa)	CCT	Color coordinate	
		x	*y*
0	1799 K	0.615	0.382
0.46	1750 K	0.609	0.388
1.54	1736 K	0.606	0.39
2.47	1731 K	0.605	0.391
3.37	1717 K	0.601	0.395
4.09	1710 K	0.594	0.401
4.84	1738 K	0.583	0.412
5.67	1858 K	0.563	0.426

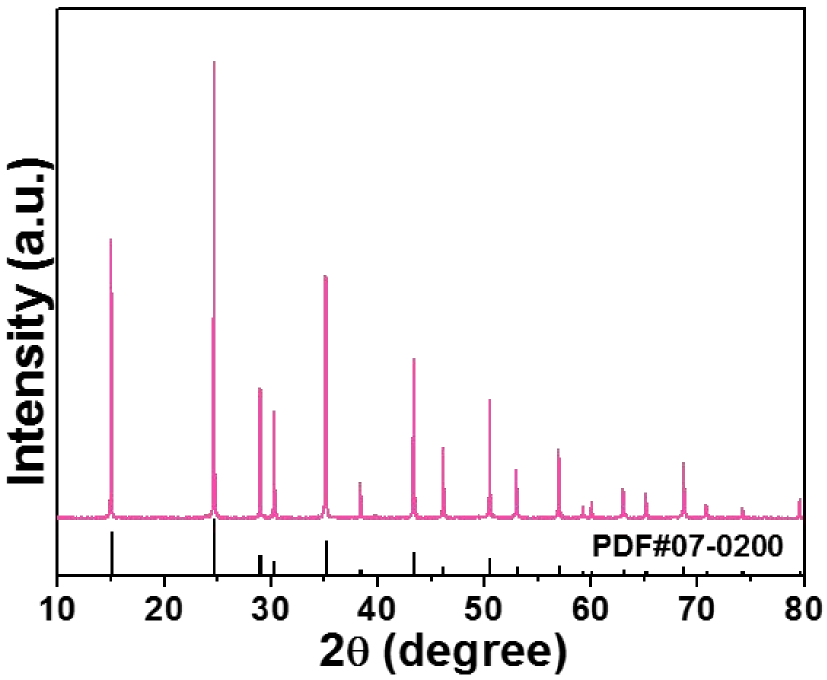

Figure S1 XRD pattern of the Cs_2PtCl_6 double perovskite.

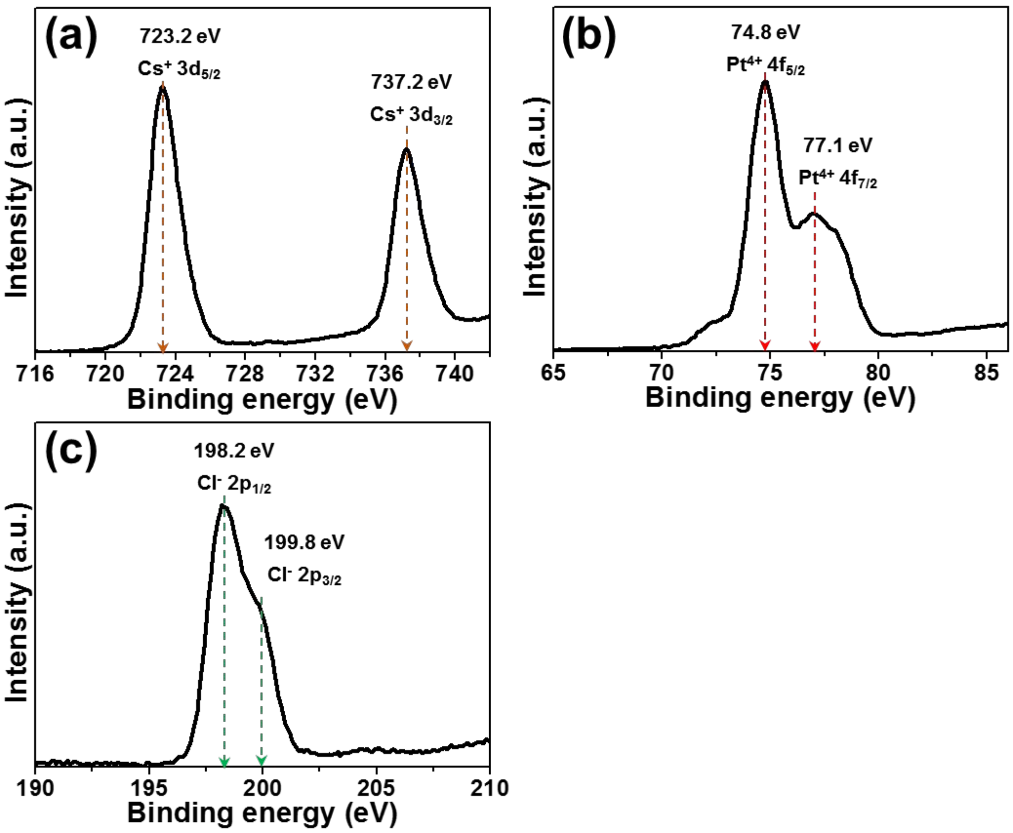

Figure S2 High-resolution XPS spectra of (a) Cs^{+} 3d, (b) Pt^{4+} 4f and (c) $C1^{-}$ 2p in the Cs_2PtCl_6 double perovskite.

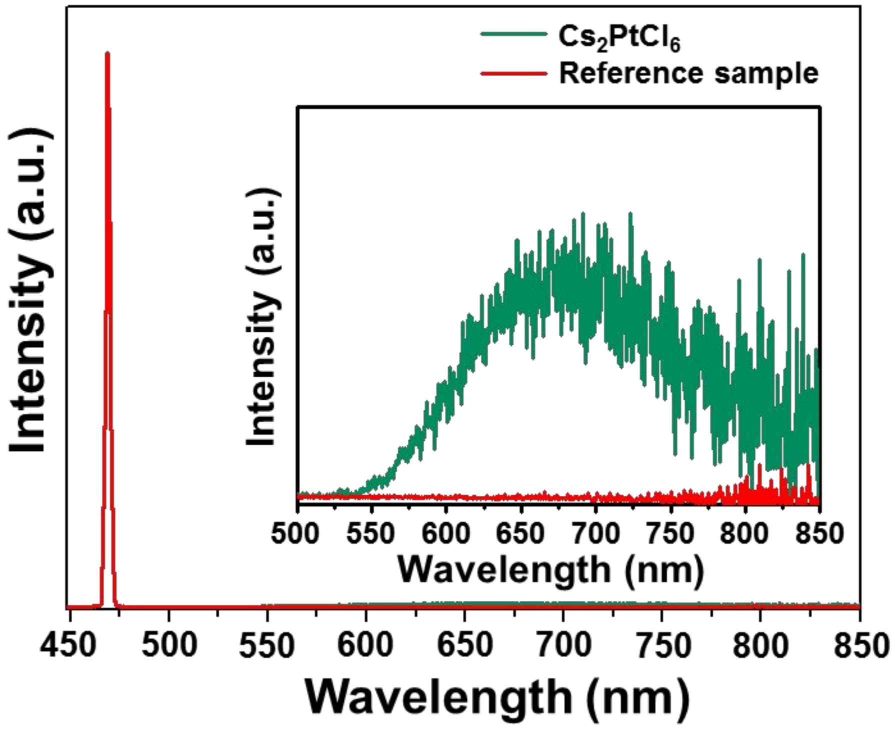

Figure S3 Quantum efficiency measurement of the Cs_2PtCl_6 double perovskite excited at 468 nm.

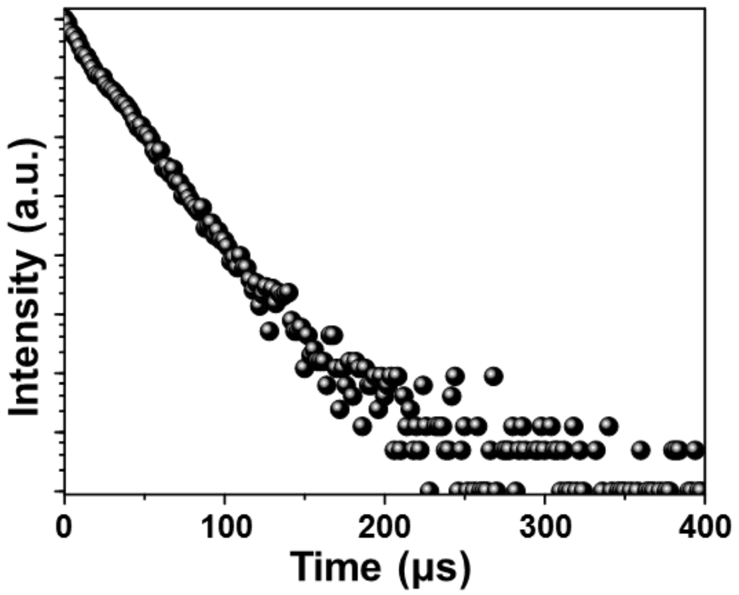

Figure S4 Decay curve of the Cs_2PtCl_6 double perovskite, where the excitation and monitoring wavelengths are 468 and 675 nm, respectively.

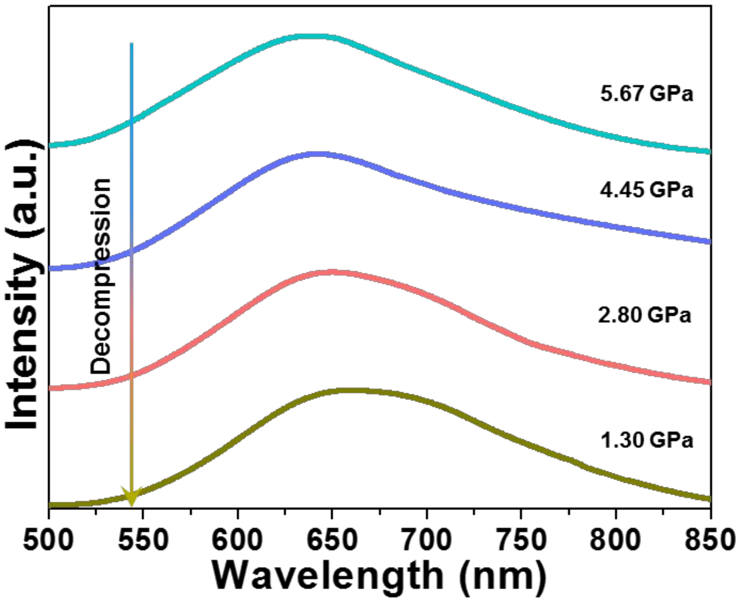

Figure S5 Pressure-dependent emission spectra of the Cs_2PtCl_6 double perovskite during the decompression process.

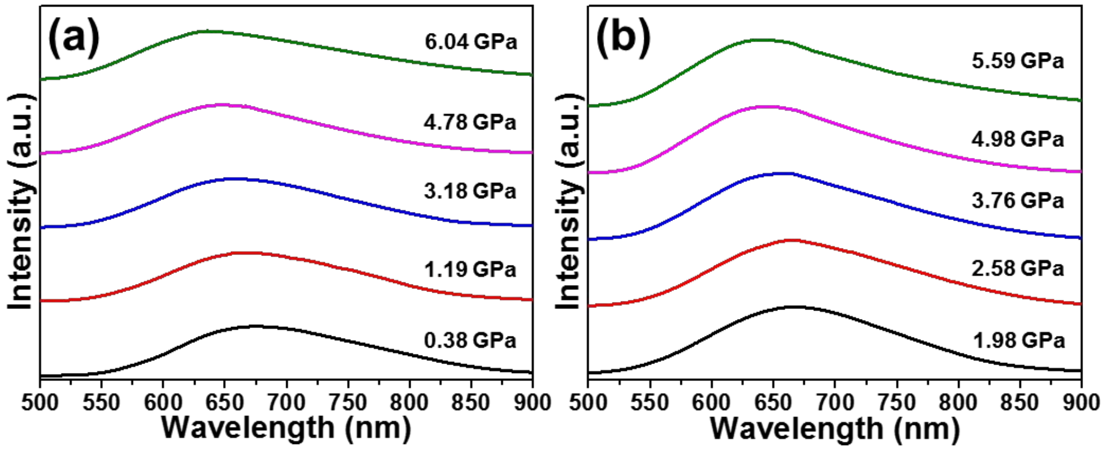

Figure S6 (a) and (b) Pressure-dependent emission spectra of the Cs_2PtCl_6 double perovskite during the compression process.
